# Could microtubule inhibitors be the best choice of therapy in gastric cancer with high immune activity: mutant DYNC1H1 as a biomarker

**DOI:** 10.18632/aging.104084

**Published:** 2020-11-20

**Authors:** Jin Bai, BoWen Yang, Ruichuan Shi, Xinye Shao, Yujing Yang, Fang Wang, Jiawen Xiao, Xiujuan Qu, Yunpeng Liu, Ye Zhang, Zhi Li

**Affiliations:** 1 Department of Medical Oncology, The First Hospital of China Medical University, Shenyang 110001, China; 2 Key Laboratory of Anticancer Drugs and Biotherapy of Liaoning Province, The First Hospital of China Medical University, Shenyang 110001, China; 3 Liaoning Province Clinical Research Center for Cancer, Shenyang 110001, China; 4 Key Laboratory of Precision Diagnosis and Treatment of Gastrointestinal Tumors, Ministry of Education, Shenyang 110001, China; 5 Department of Medical Oncology, Shenyang Fifth People Hospital, Tiexi District, Shenyang 110001, China; 6 Laboratory I of Cancer Institute, The First Hospital of China Medical University, Shenyang 110001, China

**Keywords:** immune checkpoint blockade, microtubule inhibitors, mutation, DYNC1H1, gastric cancer

## Abstract

Immune checkpoint blockade (ICB) has achieved unprecedented breakthroughs in various cancers, including gastric cancer (GC) with high immune activity (MSI-H or TMB-H), yet clinical benefits from ICB were moderate. Here we aimed to identify the most appropriate drugs which can improve outcomes in GC. We firstly compared MSI-H and TMB-H GC samples with normal samples in TCGA-STAD cohort, respectively. After that, Connectivity Map database repurposed nine candidate drugs (CMap score < -90). Then, microtubule inhibitors (MTIs) were screened as the significant candidate drugs with their representative gene sets strongly enriched (*p* < 0.05) via GSEA. GDSC database validated higher activities of some MTIs in GC cells with MSI-H and TMB-H (*p* < 0.05). Furthermore, some MTIs activities were positively associated with mutant Dynein Cytoplasmic 1 Heavy Chain 1 (DYNC1H1) (*p* < 0.05) based on NCI-60 cancer cell line panel. DYNC1H1 was high frequently alteration in GC and was positively associated with TMB-H and MSI-H. Mutant DYNC1H1 may be accompanied with down-regulation of MTIs-related genes in GC or change the binding pocket to sensitize MTIs. Overall, this study suggested that some MTIs may be the best candidate drugs to treat GC with high immune activity, especially patients with DYNC1H1 mutated.

## INTRODUCTION

High immune activity is one of the essential characteristics of malignant tumors [1]. Currently, as the advent of immune checkpoint blockade (ICB), GC patients with microsatellite instability-high (MSI-H) exhibited sensitivity to ICB in multiple clinical trials [2–4]. Tumors with high tumor mutational burden (TMB-H) also showed better responses to ICB and more prolonged overall survival than those with low TMB in gastric cancer [5]. Therefore, MSI-H and TMB-H can be considered as the high immune activity biomarkers in GC to repurpose better drugs that can improve clinical outcomes.

ICB has achieved overwhelming breakthroughs in the treatment of different malignant tumors such as GC [6, 7]. ICB significantly prolonged the overall survival of GC patients [2, 4, 8, 9]. However, the therapeutic benefit was limited to specific subgroups such as MSI-H and TMB-H and so on. Therefore, ongoing studies are underway to improve the efficacy of ICB through the following two main ways: 1) selecting patients with high immune activity subgroups for ICB treatment [5]; 2) in combination with conventional chemotherapy regimens or other drugs [10, 11]. However, there were still no standard recommendation for the combination drugs. Hence, we considered it valuable to study that the drugs which target the high immune activity subgroups may be a more appropriate choice.

Currently, it is difficult to obtain population data in the short term. High-throughput databases of pharmacogenomics are practical approaches for preliminary drug repurposing. For instance, the Connectivity Map (CMap) database provides drug-induced gene expression profiles from 72 cell lines by 27,927 compounds [12]. This database has been used to identify rapamycin for improving the prognosis of GC [13]. Another study reported that ERCC1 and DPD might be the oxaliplatin-resistant genes, based on the NCI-60 cancer cell line panel [14, 15]. Another study constructed a methodology of determining the optimal combination of chemotherapy drugs for gastric cancer patients based on chemotherapeutic drug responses to DCF from the genomics of drug sensitivity in cancer (GDSC) database [16, 17].

In the current study, we selected TCGA-STAD (Stomach Adenocarcinoma) patients with high immune activity (MSI-H, and TMB-H) as the research objects. We then used online omics tools such as CMap, NCI-60, GDSC databases to identify candidate drugs that could more potential for the treatment of GC with high immune activity as well as the predictive biomarker for the drug sensitivity of candidate drugs and possible mechanisms.

## RESULTS

### Identification of differentially expressed genes (DEGs) in gastric cancer with high immune activity

To identify DEGs in GC with high immune activity, we used the GC (N = 375) and adjacent normal tissues (N = 32) of the TCGA-STAD cohort. 12.5% (47/375) GC samples displayed MSI-H, and 15.7% (118/375) of GC patients were TMB-H. Indeed, DEGs were screened from each group compared with normal tissues. Overall, a total of 539 up-regulated and 1353 down-regulated genes were found in the MSI-H group, 632 up-regulated and 979 down-regulated genes were screened from TMB-H group. Together, there were 1375 common DEGs in the two groups: 436 common up-regulated and 939 common down-regulated genes (common DEGs were shown in Supplementary Table 1).

### Identification of high immune activity targeting candidate drugs for gastric cancer

**Figure 1:**
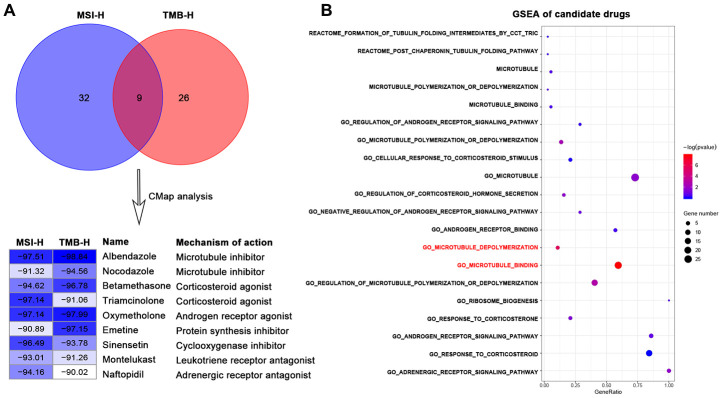
**Identification of candidate drugs that may treat gastric cancer with high immune activity in TCGA-STAD cohort.** (**A**) Venn diagram (top) showing the number of common candidate drugs (CMap score < -90) from MSI-H (blue) and TMB-H (red) groups via the connectivity map database. Connectivity score table (down) displaying nine common candidate drugs, each row responsible for a drug and columns corresponding to MSI-H and TMB-H groups. The score labels representing the connectivity score of each drug in each group, and right sides of the table indicating the name and mechanism of each drug. These nine drugs can be classified into seven types. (**B**) GSEA results for candidate drugs based on the functional gene sets of these seven drug types from the Molecular Signatures Database by the function of enricher of clusterprofiler package. Pathways in red font were significantly enriched (*p* value < 0.05).

We firstly used DEGs of MSI-H and TMB-H groups to query in the CMap database respectively to repurpose candidate drugs. CMap results revealed nine drugs both from the two groups were significantly negatively correlated with the DEGs (connectivity score <-90). These nine drugs can be classified into seven types (Figure 1A) according to the annotation information of the CMap database and previous studies [18–23].

We next sought to the significant candidate drugs. We screened the representative functional gene sets of the seven drug types from the Molecular Signatures Database (MSigDB) (Supplementary Table 2). Based on the common DEGs, GSEA was performed with representative functional gene sets for each drug type. GSEA results showed that some microtubule inhibitors-related pathways: "GO_MICROTUBULE_BINDING", "GO_MICROTUBULE_DEPOLYMERIZATION" were significantly enriched (*p*-value < 0.05) in DEGs of high immune activity, while other types of drug showed no statistical significance (*p*-value > 0.05) (Figure 1B).

**Figure 2:**
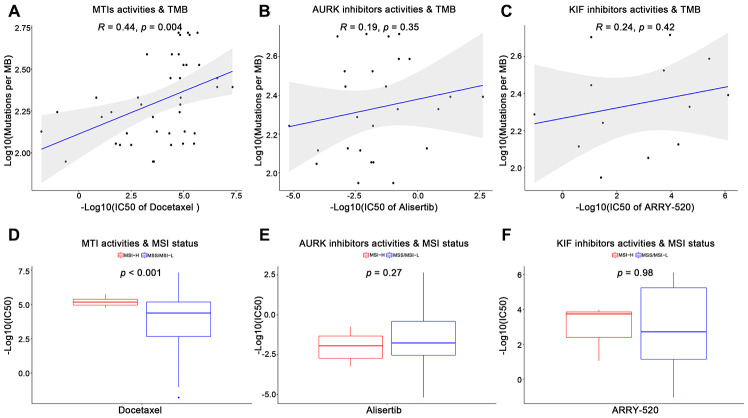
**Validation of microtubule inhibitors (MTIs) in gastric cancer cell lines with high immune activity based on GDSC database.** (**A**–**C**) Plot depicting the correlations of IC50 values of some tubulin related drugs ((**A**) MTIs, (**B**) AURK inhibitors, (**C**) KIF inhibitors) with TMB levels in GC cell lines based on GDSC database using Spearman’s correlation. (**D**–**F**) Histograms showing the different levels of IC50 values of some tubulin related drugs ((**D**) MTIs, (**E**) AURK inhibitors, (**F**) KIF inhibitors) between MSI-H and MSS/MSI-L GC cell lines based on GDSC database using Student’ s t test. *P*-value < 0.05 was considered significant.

Furthermore, we utilized the genomics of drug sensitivity in cancer (GDSC) database to validate the anticancer sensitivities of MTIs in GC with high immune activity. A total of 13 tubulin related drugs (such as MTIs, AURK inhibitors, and KIF inhibitors) and 26 gastric cancer cells were available in the GDSC database. For the TMB levels and MSI status in GC cell lines, some MTIs showed significant associations with TMB levels (Figure 2A, docetaxel: R = 0.44, p-value = 0.004) and MSI-H (Figure 2D, docetaxel: p-value < 0.001). However, no significance was found in other tubulin related drugs, including AURK inhibitors (Figure 2B, 2E, alisertib: p-values > 0.05) and KIF inhibitors (Figure 2C, 2F, ARRY-520: p-values > 0.05) (complete results were shown in Supplementary Table 2).

Moreover, survival analysis revealed three MTIs related genes (like BUB1B) in TMB group and 12 MTIs related genes (like ABCG2) in MSI-H group had significant prognostic values (Supplementary Figure 1). Together, we referred that some MTIs were more likely to treat GC with high immune activity.

### Prediction of the mutant gene for candidate drugs activity and its association with high immune activity

**Figure 3:**
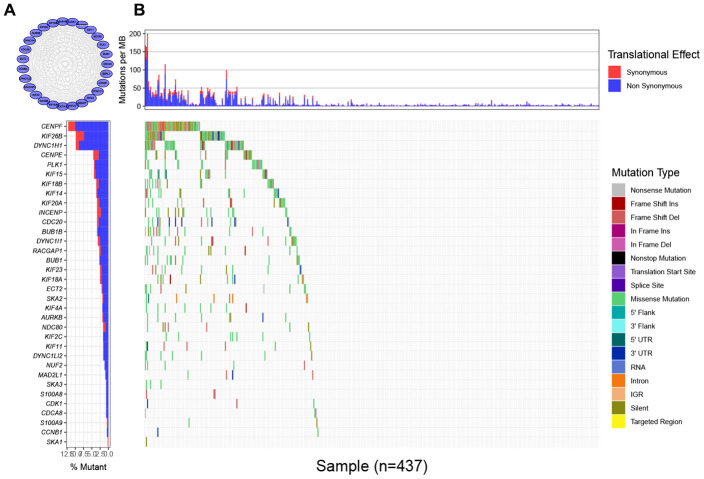
**Mutational landscape of genes involved in pathways of microtubule inhibitors (MTIs) in TCGA-STAD cohort.** (**A**) Protein-protein interaction (PPI) network of genes that significantly enriched in microtubule inhibitors (MTIs) representative gene sets. (**B**) Mutational landscape of node genes from PPI network showing that CENPF, KIF26B, and DYNC1H1 were highly mutated in TCGA-STAD cohort (alteration frequency ≥ 7%) by the GenVisR package. Top for somatic mutation rate of each sample, bottom left for the total mutation frequency of each gene, and bottom right for specific mutation type of each gene in each sample.

To find the characteristic gene which could suggest the activities of MTIs in GC with high immune activity, we started with a PPI network analysis. Based on the 17 common DEGs directly enriched in the MTIs representative pathways, we constructed a PPI network with 30 nodes and 289 edges (Figure 3A). After that we carried out a mutational landscape analysis on these 30 node genes and revealed that three genes (DYNC1H1, KIF26B, and CENPF) were mutated at a high frequency (mutation frequency ≥ 7%) in the TCGA-STAD cohort (Figure 3B).

**Figure 4:**
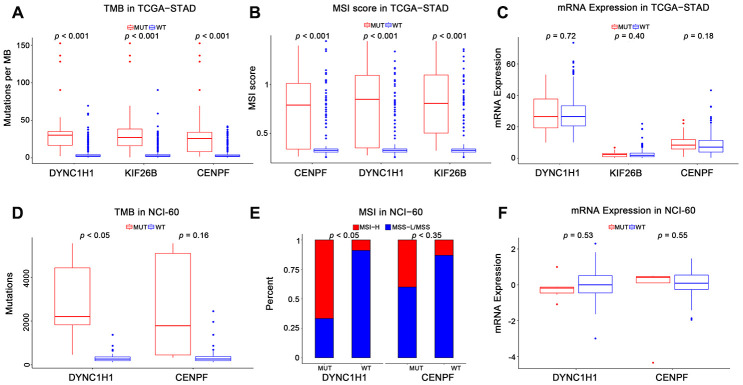
**Association of mutant genes with high immune activity in TCGA-STAD and NCI-60 cell lines datasets.** Levels of TMB, MSI score, and mRNA expression in TCGA-STAD (**A**–**C**) and NCI-60 cell lines (**D**–**F**), stratified by genes (DYNC1H1, CENPF, and KIF26B) mutation status. MUT: mutated, WT: wild type. KIF26B was not detected in NCI-60 cell lines dataset. All *p-*values were obtained by Student’s t-test, in addition to χ^2^ test for (**E**). *P*-value < 0.05 was considered significant.

To characterize the roles of these three genes in high immune activity, we separately compared the levels of TMB, MSI score, and mRNA expression between these three genes mutated and wild type tumors based on the TCGA-STAD, NCI-60 cell lines and immunotherapy datasets. As shown in Figure 4 and Supplementary Figure 2, mutations in DYNC1H1 were significantly correlated with TMB-H and MSI-H in these above datasets. Mutations in CENPF were considerably correlated with TMB-H and MSI-H in the TCGA-STAD cohort, but not in the NCI-60 cell lines or immunotherapy dataset. Mutations in KIF26B were significantly correlated with TMB-H and MSI-H in the TCGA-STAD cohort, but not immunotherapy datasets (KIF26B was not detected in NCI-60 cell lines dataset). Besides, there was no significant difference of mRNA expression levels among three genes mutated and wild type tumors in TCGA-STAD or NCI-60 cell lines dataset (Figure 4C, 4F). Together, these results indicated that mutant DYNC1H1 was strongly associated with enhanced tumor immune activity.

Furthermore, we investigated the importance of mutant DYNC1H1 in TCGA pan-cancer, showing that the mutation frequency of DYNC1H1 was 0.4%-19.7%, average mutation frequency was 5.17% in 15 cancers. DYNC1H1 mutated tumors were associated with higher TMB levels than its wild type tumors (Supplementary Figure 3A). Accordingly, the mutation frequency of DYNC1H1 was positively associated with TMB levels in 9 cancers (Supplementary Figure 3B). Moreover, DYNC1H1 was highly mutated in Uterine Corpus Endometrial Carcinoma (UCEC) and GC. There were more than 26 missense mutation spots of DYNC1H1 in GC (Supplementary Figure 3C, 3D) These results showed that the mutant DYNC1H1 was consistent with high immune activity of TMB levels in multiple cancers.

### Association of mutant DYNC1H1 with enhanced microtubule inhibitors activities

**Figure 5:**
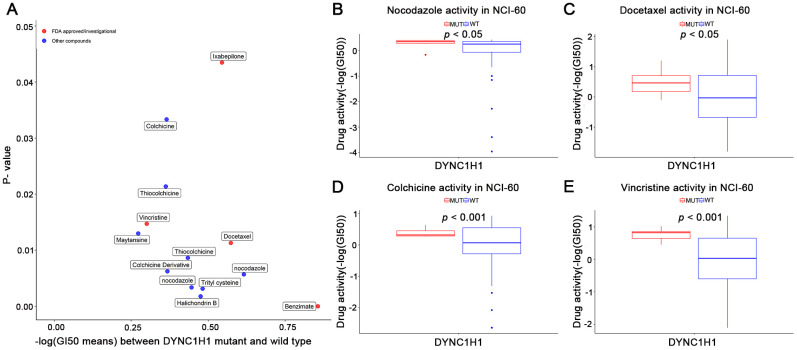
**Association of mutant DYNC1H1 with enhanced microtubule inhibitors (MTIs) activities in NCI-60 cell lines dataset.** (**A**) Volcano plot for the different activities of MTIs between DYNC1H1 mutated and wild type NCI-60 cell lines dataset. The x-axis represented the different levels of mean -logGI50, and the y-axis showed *p*-values obtained by Student’s t-test. (**B**–**E**) Histograms depicting different MTIs activities of nocodazole (**B**), docetaxel (**C**), colchicine (**D**), vincristine (**E**), stratified by DYNC1H1 mutation status in NCI-60 cell lines dataset (*p*-value < 0.05 by Student’s t-test). MUT: mutated, WT: wild type. *P*-value <0.05 was considered significant. DYNC1H1 was mutated in six NCI-60 cell lines: HCC_2998, HCT_116, HCT_15, KM12, MOLT_4, and UACC_62.

To explore the interaction between mutant DYNC1H1 and MTIs, we firstly selected 41 MTIs included in NCI-60 cell lines dataset. 13 of these MTIs have significantly increased activities in the DYNC1H1 mutated cell lines (Figure 5, *p*-value < 0.05). Furthermore, a clear trend of higher MTIs activities was observed in DYNC1H1-mutated GC cells (Supplementary Figure 4). These results suggested that mutations in DYNC1H1 may be an essential biomarker for the antitumor effects of MTIs.

### Effects of mutant DYNC1H1 on enhanced microtubule inhibitors activities

**Figure 6:**
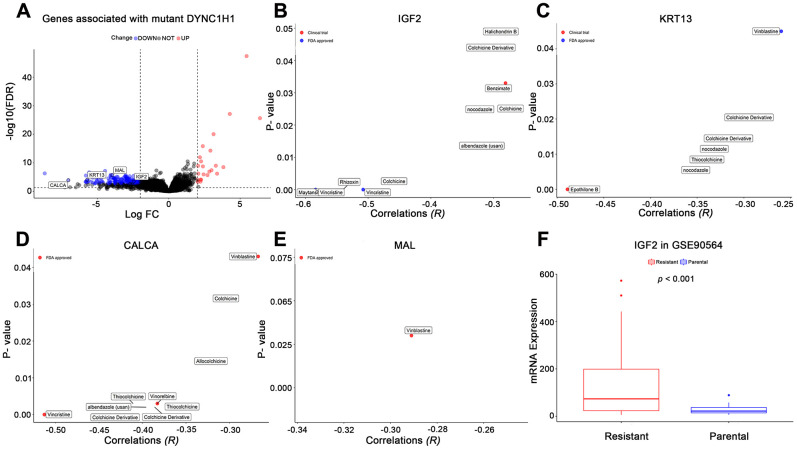
**Effects of mutant DYNC1H1 on enhanced microtubule inhibitors (MTIs) activities.** (**A**) Volcano plot for the DEGs between DYNC1H1 mutated and wild type gastric cancer patients in TCGA-STAD cohort. The x-axis represented log2 (fold change): mutant DYNC1H1 compared with wild type patients, and the y-axis represented significant difference as −log10(FDR). The criteria of FDR <0.05 and |log2FC| ≥ 2 were considered significant by the function TCGAbiolinks_DEA of TCGAbiolinks. (**B**–**E**) Correlations between MTIs activities and mRNA expressions of IGF2, KRT13, CALCA, and MAL in NCI-60 cell lines dataset, respectively. *P*-value estimated using Pearson’s correlation. The x-axis represented the correlation coefficient, and the y-axis showed the significance (−log10 p-value). (**F**) Histograms depicting different mRNA expressions of IGF2 between MTIs sensitive and resistant cell lines in GSE90564 dataset. *P*-value estimated using Student’s t-test. *P*-value <0.05 was considered significant.

To further explore how mutant DYNC1H1 increase drug sensitivities of MTIs, we used a differential analysis method to identify mutant DYNC1H1 related genes by comparing DYNC1H1 mutated tumor with wild type tumors (37/338) in TCGA-STAD. A total of 199 genes were significantly correlated with mutant DYNC1H1 (23 up-regulated and 176 down-regulated genes) (Figure 6A). Based on NCI-60 cell lines dataset, the expressions of four genes (IGF2, MAL, KRT13, CALCA) were negatively associated with MTIs sensitivities (Figure 6B–6E, *p*-value < 0.05). Accordingly, IGF2 expression levels were lower in paclitaxel sensitive breast cancer cells than in paclitaxel resistant breast cancer cells in GSE90564 (Figure 6F, *p*-value < 0.05). Together, these results suggested that the process of mutant DYNC1H1 sensitizing MTIs may be followed by decreased expressions of MTIs related genes such as IGF2, KRT13, CALCA and MAL.

## DISCUSSION

**Figure 7:**
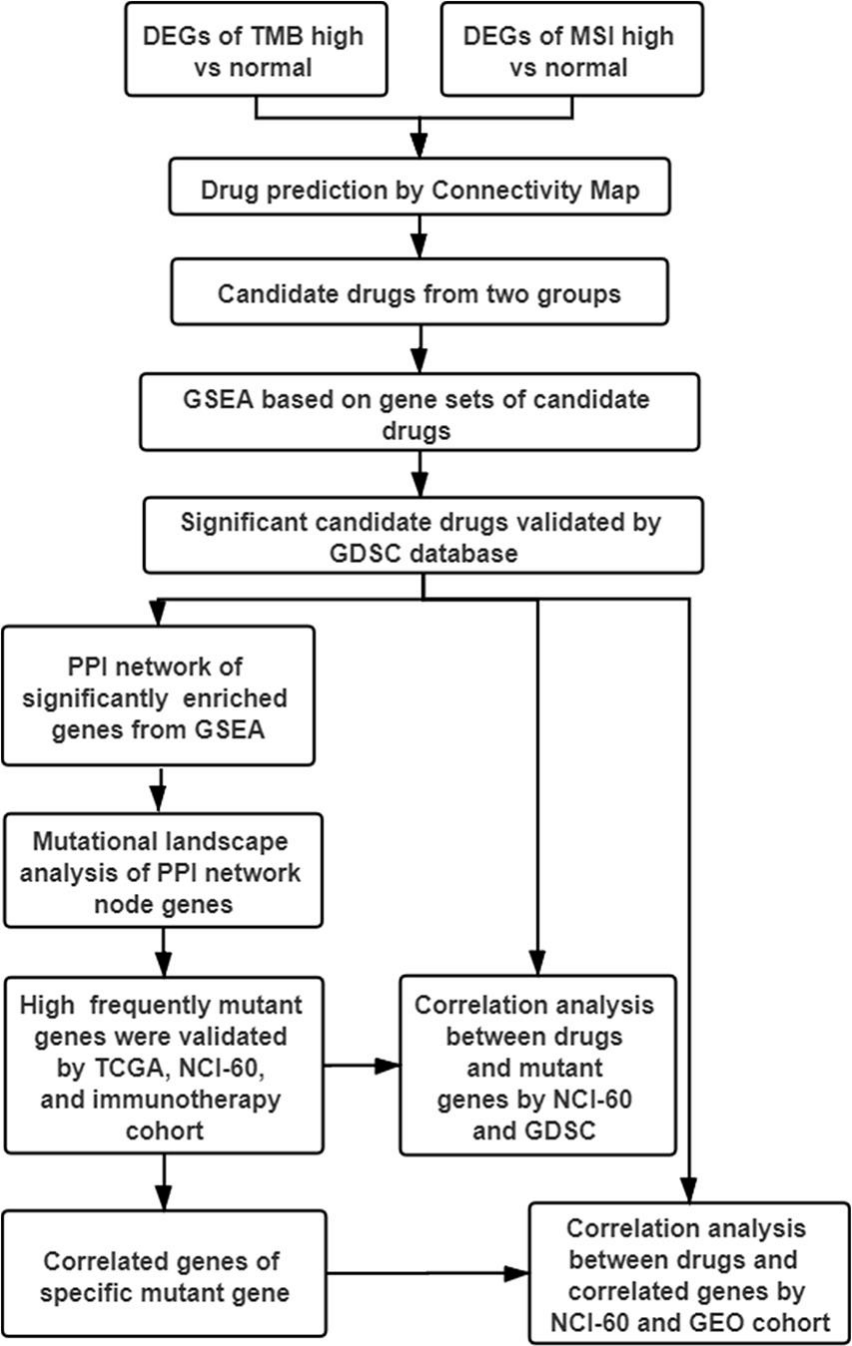
**Work flow of the study.** DEGs: differentially expressed genes; TMB-H: high tumor mutational burden; MSI-H: high microsatellite instability; GSEA: gene sets enrichment analysis; GDSC: genomics of drug sensitivity in cancer; PPI: protein-protein interaction; TCGA: The Cancer Genome Atlas; NCI-60: National Cancer Institute 60.

The advent of cancer immunotherapy, such as the approval of anti-PD1 monoclonal antibodies, has altered the treatment paradigm of many malignancies including GC [6, 7]. However, the clinical benefits were quite low and limited to high immune activity subtypes patients [2–5]. In this study, MTIs were repurposed as the more appropriate drugs to treat GC with high immune activity (TMB-H and MSI-H) based on CMap database and GSEA (Figure 7). Then, MTIs like docetaxel showed increased activities when the mutations in DYNC1H1. Furthermore, mutant DYNC1H1 can act as a biomarker for MTIs activities possibly because it may be followed by lower levels of MTIs-related genes, or some mutations in DYNC1H1 may change the binding confirmation of MTIs and microtubules. In summary, our research indicated that GC patients with high immune activity may benefit from some MTIs that can be sensitized by mutant DYNC1H1.

This study presented that some MTIs may treat high immune activity subgroup GC patients by several lines of evidence. Firstly, nocodazole and albendazole were recognized as MTIs from CMap database (Figure 1A). Nocodazole can affect the dynamics of microtubules by specifically binding to the cell motility apparatus [18]. Apart from the indicator of an anthelmintic benzimidazole carbamate [19, 20], albendazole also can inhibit microtubule polymerization for the treatment of gastric cancer and other cancers [21–23]. Then, some MTIs representative pathways can be significantly enriched in high immune activity of GC: "GO_MICROTUBULE_BINDING", "GO_MICROTUBULE_DEPOLYMERIZATION", whilst other drug types repurposed by CMap showed no significance (Figure 1B). Moreover, the activities of some MTIs (such as docetaxel) were strongly higher in MSI-H and TMB-H gastric cancer cells in the GDSC database. However, activities of other tubulin related drugs (such as AURK inhibitors, KIF inhibitors) revealed no significance in different MSI status and TMB levels (Figure 2).

MTIs are widely known for binding to the microtubules [24, 25]. Among then, taxanes (paclitaxel and docetaxel) have shown antitumor activity in the treatment of GC according to the National Comprehensive Cancer Network (NCCN) guidelines. In addition to antiproliferative effects, growing studies have reported that some MTIs can be immunostimulatory properties, including their abilities to reprogram the immune-suppressive M2 profile of tumor-associated macrophages to immune-stimulating M1 profile [26], stimulate maturation and activities of dendritic cells [27, 28], and decrease the accumulation and immunosuppressive activities of tumor-infiltrating MDSCs [29]. Clinically, single-agent paclitaxel even showed a better median progression-free survival than the anti-PD1 pembrolizumab (4.1 months vs 1.5 months) in the KEYNOTE-061 clinical trial (PDL1 CPS>1) [4]. These evidences supported that some MTIs may achieve good outcomes in gastric cancer patients with high immune activity.

Our results clearly demonstrated that drug activities of some MTIs were strongly associated with mutant DYNC1H1 (Figure 6). DYNC1H1 is the heavy chain of cytoplasmic dynein which acts as a motor protein using ATP to travel along the microtubule (MTs) toward minus end [30, 31]. This complex participates in multiple cell processes, such as spindle formation in mitosis and transportation of various cellular cargoes. Mutant DYNC1H1 was reported to impede the ATP hydrolysis cycle which help bind to MTs in the neurological diseases [32]. Most mutations of DYNC1H1 were tied up with the occurrence and development of pancreatic cancer [33–35], suggesting that mutations in DYNC1H1 may play a vital role in the complex biological process of malignant tumors. However, the effect of its mutation on gastric cancer remains unknown.

In this study, mutant DYNC1H1 was closely associated with MTIs-related genes (IGF2, KRT13, MAL, and CALCA) (Figure 5). IGF2 and anti-apoptotic gene KRT1 were significantly overexpressed in MTIs- resistant cancer cells, and exhaustion of IGF2 can restore paclitaxel sensitivity [36, 37]. Also, CALCA and MAL were reported to be highly expressed in the allergic reactions of MTIs [38, 39]. On the other hand, DYNC1H1 directly binds to MTs via the structure called MTBD [40, 30]. Besides, MTIs (like paclitaxel and docetaxel) exert antitumor effects through binding to β-tubulin [41, 42]. Hence we speculated that mutant DYNC1H1 may narrow the binding pocket of MTBD and β-tubulin and then increase binding pocket of MTIs and β-tubulin, thereby enhancing the anti-microtubule effect of MTIs. We identified 26 missense mutation sites of DYNC1H1 in the TCGA-STAD mutation profile (Supplementary Figure 3). However, there were no studies clearly illustrated the correlation of these mutation sites of DYNC1H1 with gastric cancer.

Our study has some limitations such as few normal samples included in this study but no other suitable datasets available for validation. We cannot also rule out the possibility that other drugs or combination therapy may show more benefits for GC patients with high immune activity. Therefore, we plan to use our own clinical samples for verification analysis in the future. Further investigation of how mutant DYNC1H1 sensitizes MTIs will be necessary in future studies.

To conclude, our study identified some MTIs such as docetaxel that could potentially be the best drugs for GC with high immune activity (TMB-H and MSI-H). Mutant DYNC1H1 significantly positively correlated with TMB-H and MSI-H in GC or various cancers. We found that mutant DYNC1H1 can sensitize MTIs, possibly because it was accompanied with down-regulation of some MTIs resistant or side effect genes. It may also change the binding pocket of MTIs and microtubules.

## MATERIALS AND METHODS

### Gastric cancer dataset and differentially expressed genes (DEGs) associated with high immune activity

GC dataset was acquired from GDC Data Portal TCGA-STAD (n = 407). "Level 3" RNA sequencing data (raw count) and clinical information were downloaded by using R package TCGAbiolinks [43]. Then, patients were classified into two groups: TMB-H group (median as cut-point of tumor mutational rate), and MSI-H group. Finally, differential analysis of the two groups were both used by the function TCGAbiolinks_DEA. DEGs were determined with the criteria of |log2(FC)| > 2 and FDR < 0.05.

### Drug prediction

The CMap database (https://clue.io/cmap) was used to identify drugs that can effectively treat high immune activity subgroups GC. Input data require a range of 10 ~ 150 genes that are up-regulated and/or down-regulated. Because the number of DEGs in the two groups of TMB-H and MSI-H were more than 150, we selected top150 up-regulated DEGs following the FDR in ascending order. A so-called Connectivity Score (-100 ~100) is an indicator for evaluating the correlation between a drug and input genes. The score less than -90 can be considered that the drug is significantly negatively related to the input genes, that is, the drug can dramatically reverse the role of these genes to treat specific diseases. Drugs correlated with the input genes of TMB-H and MSI-H group were selected as candidate drugs for subsequent analysis.

### Gene sets enrichment analysis (GSEA)

GSEA was used to further screen the significant candidate drugs. Candidate drugs-related gene sets were selected from MSigDB [44], and common DEGs were used as input data. GSEA was performed separately with gene sets of each drug type using the function enricher of clusterprofiler [45] package. The drug corresponding to the significantly enriched gene sets (*p*-value ≤ 0.05) was regarded as significant candidate drug.

### Gastric cancer data of drug sensitivity and high immune activity

To validate the role of significant candidate drugs for treating gastric cancer with high immune activity, we extracted the half maximal inhibitory concentration (IC50) values of them in GC cell lines from GDSC (https://www.cancerrxgene.org/downloads/anova) database. MSI status of each GC cell line was also provided by GDSC. TMB levels of each cell line were retrieved from the CCLE (https://portals.broadinstitute.org/ccle/data) database.

### Protein-protein interaction (PPI) network

Search Tool for the Retrieval of Interacting Genes (STRING; string-db.org) was used to build a PPI network for genes directly enriched in gene sets of significant candidate drugs. The parameter of interaction was set as the interaction score > 0.9 and no more than 20 interactors. Cytoscape software 3.7.0 was used to visualize the PPI network.

### Mutational landscape analysis

Somatic mutational landscape analysis was used to explore characteristic genes of significant candidate drugs. Mutational profile of TCGA-STAD was obtained from TCGA data portal by the GDCquery_Maf function of the TCGAbiolinks package in R. Using "GenVisR" package to process and visualize the mutational burden of PPI network node genes. The specific mutant genes were identified with mutation frequency ≥ 7% [46].

### Data of gene mutation and high immune activity

To compare different levels of high immune activity (TMB, MSI) and mRNA expression between specific mutant genes mutated and wild type tumors. TCGA-STAD, NCI-60, immunotherapy cohort (Allen cohort), and TCGA pan-cancer datasets were selected.

For TCGA-STAD dataset, gene mutational profile and mRNA expression data, TMB levels (tumor mutational rate), MSI score [47] data were used. For NCI-60 cancer cell lines dataset, binary gene mutation and mRNA expression data were available from R package rcellminer [14], TMB levels of each cell line were downloaded from cbioportal (https://www.cbioportal.org) database, and microsatellite status data were downloaded from cosmic (https://cancer.sanger.ac.uk/cosmic#) database. For Allen cohort, consisting of 110 advanced-stage melanoma patients treated with anti-CTLA-4 therapy, provided complete somatic mutation profile [48]. For TCGA pan-cancer, mutational profiles were obtained from the TCGA data portal by the GDCquery_Maf function of the TCGAbiolinks package in R. TMB levels were calculated based on non-synonymous data. Mutation frequencies of specific mutant genes in each cancer type were estimated respectively.

### Data of drug sensitivity and gene mutation

To verify the relationship between specific mutant genes and significant candidate drugs, we used genomics data and drug sensitivity of NCI-60 cell lines dataset and GDSC database. For NCI-60 cell lines, the concentration of drug was presented as cause 50% growth inhibition (GI50) value and was available from R package rcellminer. Gene mutation information was described above Gastric cancer data of drug sensitivity and high immune activity. For GDSC database, gene mutation information was downloaded from the GDSC website (https://www.cancerrxgene.org/downloads). IC50 values of candidate drugs were described above. Data of gene mutation and high immune activity.

### The effect of specific mutant genes

To investigate the impact of specific mutant genes on significant candidate drugs. Firstly, we used differential analysis method to find genes significantly associated with the specific mutant genes. According to significant mutant genes status, DEGs were identified by the mutated group compared to the wild type group in the TCGA-STAD (the criteria of |log2 (FC)|> 2 and FDR <0.05). Secondly, to analyze the relationships between these DEGs and significant candidate drugs activities, the gene expression data and drug activity data of NCI-60 cell lines and GSE90564 (paclitaxel sensitivity and resistance dataset, n = 38) were used.

### Statistical analysis

Differential analysis was mainly conducted by the function TCGAbiolinks_DEA of TCGAbiolinks package. Survival curves were performed by the Kaplan-Meier method and compared by the log-rank test via the R package survival. Group comparisons were performed by Student’s t test for continuous variables, while χ^2^ test for categorical variables. The correlation of IC50 values of candidate drugs with TMB levels in GC cell lines of GDSC database, GI50 values of candidate drugs with gene expression or gene mutation in NCI-60 cancer cell lines dataset were identified by Spearman’s correlation analysis. All statistical tests were two-sided, and *p*-value <0.05 was considered significant. Statistical analysis was performed by R software version 3.6.1(v. 3.5.2 (http://www.r-project.org).

## Supplementary Material

Supplementary Figures

Supplementary Table 1

Supplementary Table 2

Supplementary Table 3
