# Supplementary material for: Could microtubule inhibitors be the best choice of therapy in gastric cancer with high immune activity: mutant DYNC1H1 as a biomarker
Source: Aging (Albany NY). 2020 Nov 20;12(24):25101–19. doi: 10.18632/aging.104084 (PMC7803585; doi:10.18632/aging.104084)
Supplement: Supplementary Figures [file aging-12-104084-s001.pdf]

SUPPLEMENTARY FIGURES

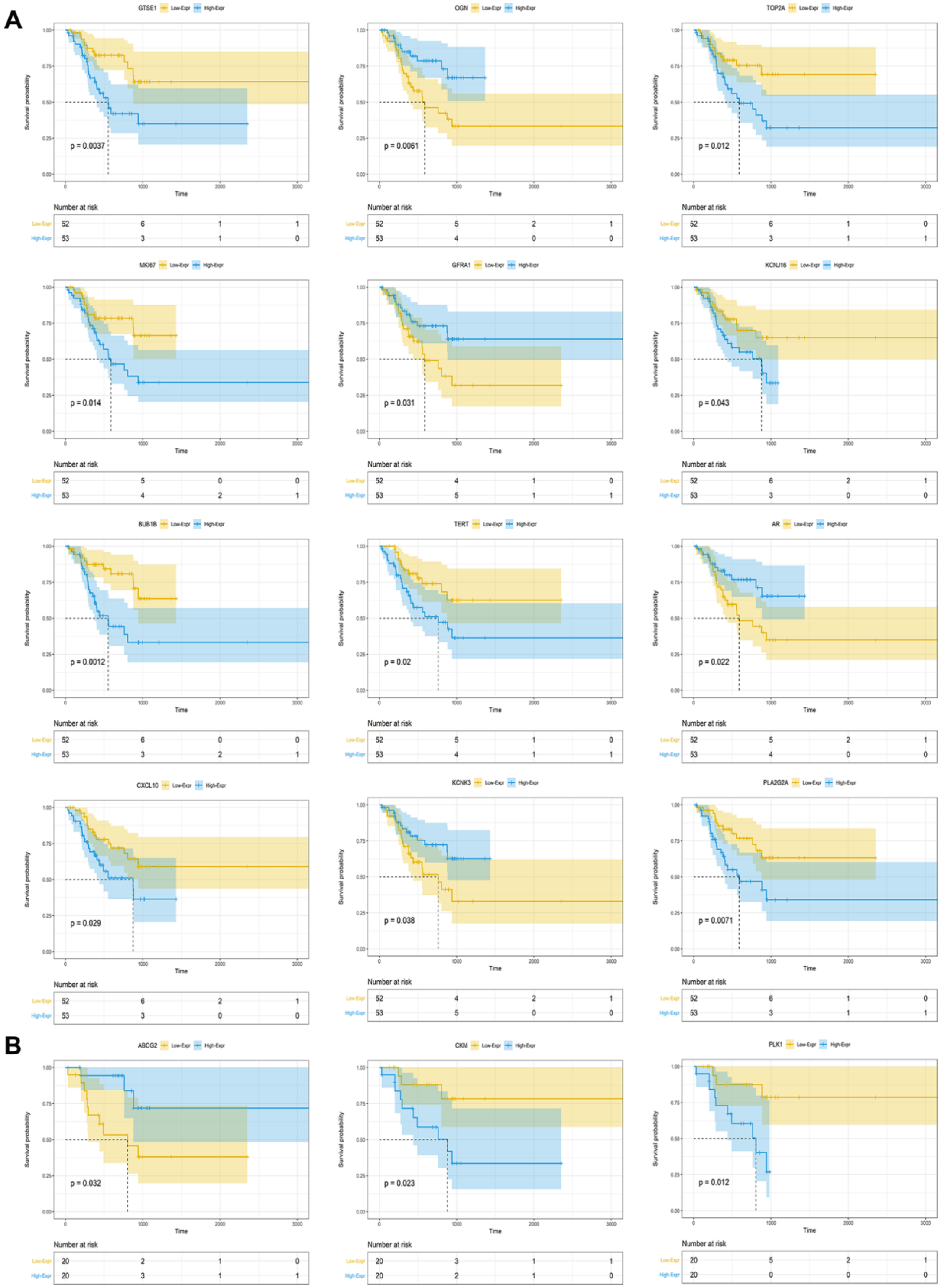

Supplementary Figure 1. The microtubule inhibitors (MTIs) related DEGs in the survival analysis of TCGA-STAD cohort. Kaplan-Meier plots for overall survival of DEGs related to MTIs in TMB-H group (**A**) and MSI-H group (**B**) by log-rank test. *P*-value <0.05 was considered significant.

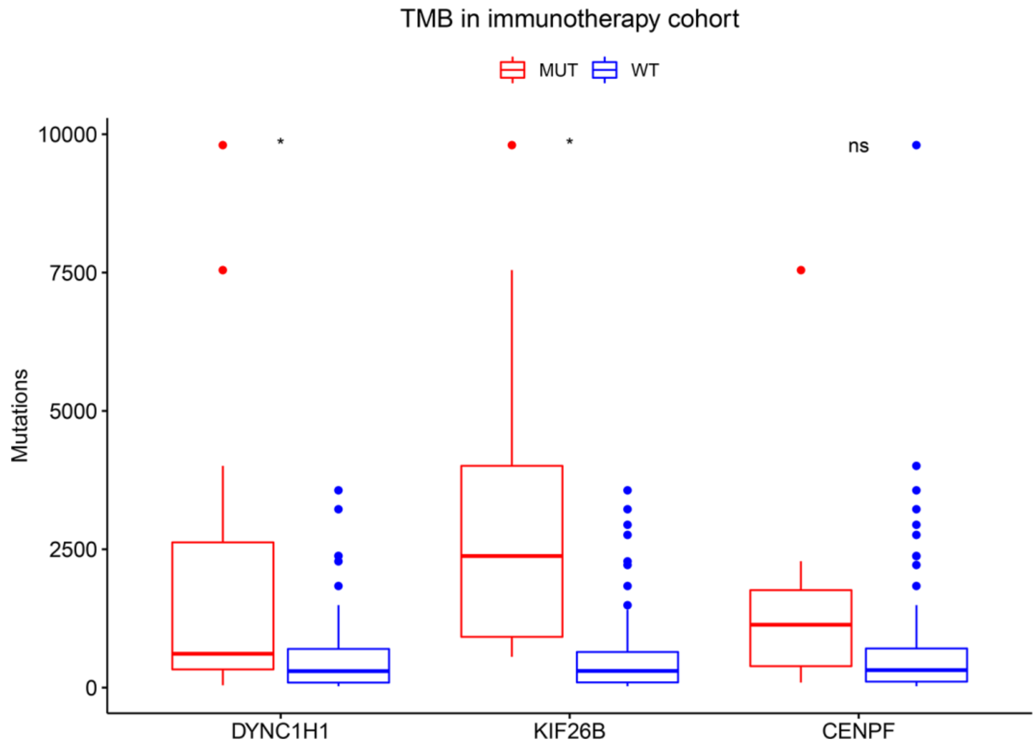

Supplementary Figure 2. Association of mutant DYNC1H1 with TMB in immunotherapy cohort. Levels of TMB in immunotherapy cohort (Allen cohort), stratified by genes (DYNC1H1, KIF26B, and CENPF) mutation status. MUT: mutated, WT: wild type. Student's t-test obtained *p*-values. ($^{*}P < 0.05$; ns, not significant).

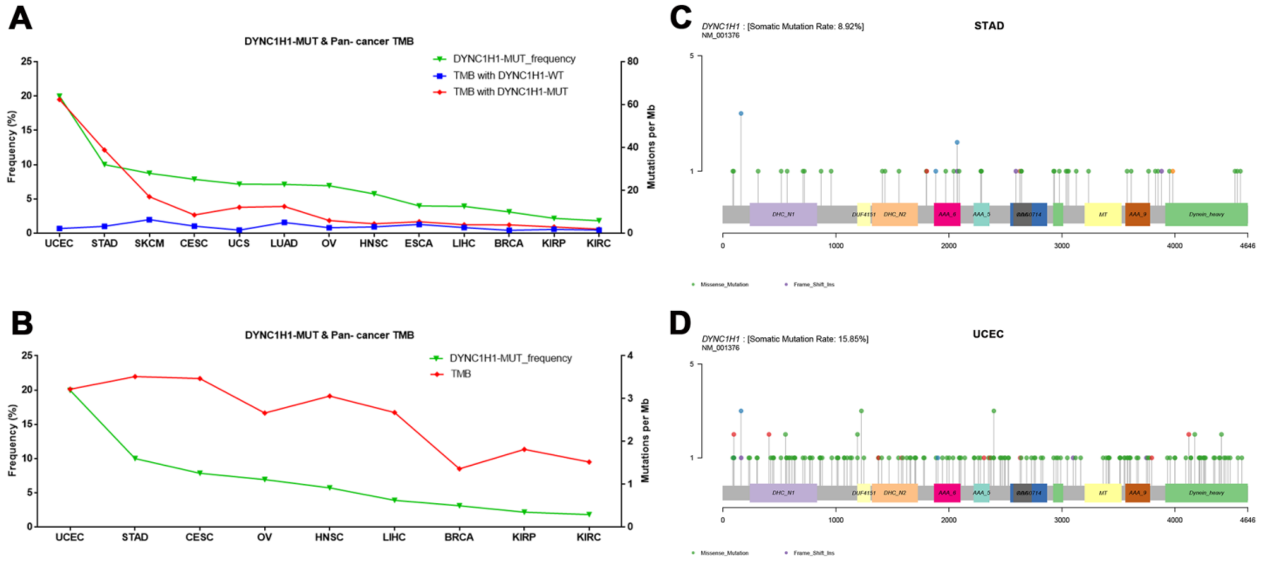

Supplementary Figure 3. Association of mutant DYNC1H1 with TMB in various cancers. (**A**) Line chart showing DYNC1H1 mutation frequency (green) and levels of TMB with DYNC1H1 mutated (red) and wild type (blue) in 15 cancers. (**B**) Line chart presenting DYNC1H1 mutation frequency (green) and levels of TMB (red) in 9 cancers. (**C**, **D**) Lollipopplots presenting the mutation spots of DYNC1H1 in gastric cancer (GC)(C) and Uterine Corpus Endometrial Carcinoma (UCEC)(**D**). Abbreviation: UCEC, Uterine Corpus Endometrial Carcinoma; STAD, Stomach Adenocarcinoma; SKCM, Skin Cutaneous Melanoma; CESC, Cervical squamous cell carcinoma and endocervical adenocarcinoma; UCS, Uterine Carcinosarcoma; LUAD, Lung Adenocarcinoma; OV, Ovarian serous cystadenocarcinoma; HNSC, Head and Neck Cancer; ESCA, Esophageal Cancer; LIHC, Liver hepatocellular carcinoma; BRCA, Breast invasive carcinoma; KIRP, Kidney renal papillary cell carcinoma; KIRC, Kidney renal clear cell carcinoma.

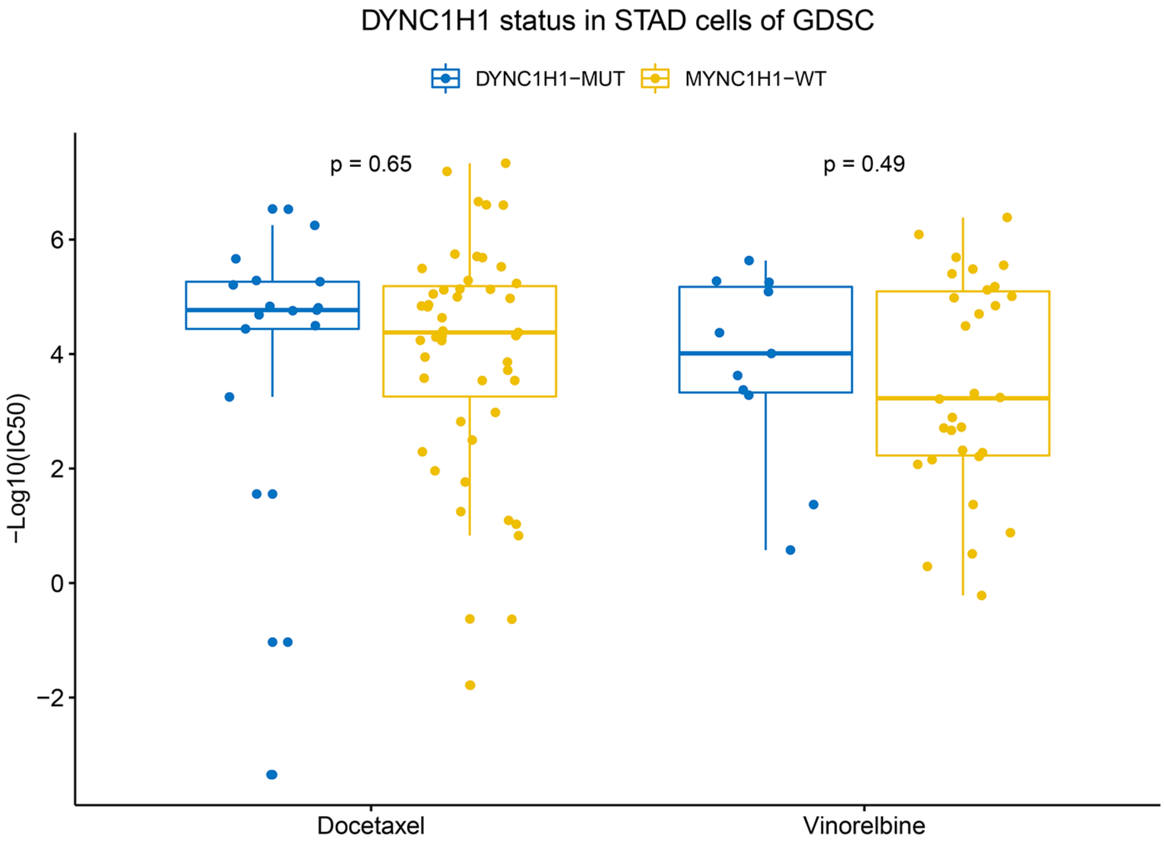

Supplementary Figure 4. Association of mutant DYNC1H1 with microtubule inhibitors (MTIs) in gastric cancer cell lines with high immune activity based on GDSC database. Histograms depicting different MTIs activities of docetaxel and vinorelbine, stratified by DYNC1H1 status in gastric cancer cell lines based on GDSC database (*p*-value < 0.05 by Student's t-test). MUT: mutated, WT: wild type.
