# Supplementary material for: Could microtubule inhibitors be the best choice of therapy in gastric cancer with high immune activity: mutant DYNC1H1 as a biomarker
Source: Aging (Albany NY). 2020 Nov 20;12(24):25101–19. doi: 10.18632/aging.104084 (PMC7803585; doi:10.18632/aging.104084)
Supplement: Supplementary Table 1 [file aging-12-104084-s002.docx]

**Supplementary Table 1. The common DEGs of MSI-H group and TMB-H group.**

| **Up-regulated-common DEGs** | **Down-regulated-common DEGs** |
| --- | --- |
| ENSG00000005073  ENSG00000005381  ENSG00000006377  ENSG00000007038  ENSG00000007350  ENSG00000007952  ENSG00000008300  ENSG00000011083  ENSG00000011426  ENSG00000013588  ENSG00000018280  ENSG00000029559  ENSG00000037965  ENSG00000038945  ENSG00000039987  ENSG00000043355  ENSG00000046774  ENSG00000049247  ENSG00000050344  ENSG00000051341  ENSG00000057468  ENSG00000058085  ENSG00000060718  ENSG00000062038  ENSG00000065328  ENSG00000066279  ENSG00000068985  ENSG00000071539  ENSG00000073067  ENSG00000075218  ENSG00000075290  ENSG00000075702  ENSG00000075891  ENSG00000077935  ENSG00000078098  ENSG00000078399  ENSG00000080511  ENSG00000081138  ENSG00000083782  ENSG00000084453  ENSG00000085999  ENSG00000087116  ENSG00000088325  ENSG00000088882  ENSG00000090889  ENSG00000091651  ENSG00000092853  ENSG00000093009  ENSG00000095627  ENSG00000095739  ENSG00000095752  ENSG00000095970  ENSG00000099399  ENSG00000099953  ENSG00000099985  ENSG00000100253  ENSG00000100473  ENSG00000100867  ENSG00000101074  ENSG00000101115  ENSG00000101197  ENSG00000101441  ENSG00000101670  ENSG00000102384  ENSG00000102854  ENSG00000103253  ENSG00000103355  ENSG00000103888  ENSG00000104327  ENSG00000104899  ENSG00000105048  ENSG00000105173  ENSG00000105219  ENSG00000105464  ENSG00000105664  ENSG00000105697  ENSG00000105989  ENSG00000106031  ENSG00000106038  ENSG00000106483  ENSG00000106484  ENSG00000106689  ENSG00000107593  ENSG00000107807  ENSG00000108688  ENSG00000108821  ENSG00000111012  ENSG00000111206  ENSG00000111247  ENSG00000111537  ENSG00000111700  ENSG00000111981  ENSG00000112984  ENSG00000113249  ENSG00000113889  ENSG00000114346  ENSG00000114854  ENSG00000115163  ENSG00000115363  ENSG00000115507  ENSG00000116014  ENSG00000117122  ENSG00000117148  ENSG00000117650  ENSG00000117724  ENSG00000118113  ENSG00000118193  ENSG00000118513  ENSG00000118785  ENSG00000119915  ENSG00000120254  ENSG00000120659  ENSG00000121075  ENSG00000121621  ENSG00000122133  ENSG00000122641  ENSG00000122861  ENSG00000123201  ENSG00000123364  ENSG00000123388  ENSG00000123407  ENSG00000123473  ENSG00000123485  ENSG00000123496  ENSG00000123500  ENSG00000123838  ENSG00000124092  ENSG00000124157  ENSG00000124233  ENSG00000124391  ENSG00000124469  ENSG00000124678  ENSG00000125207  ENSG00000125508  ENSG00000125571  ENSG00000125657  ENSG00000125726  ENSG00000126583  ENSG00000126778  ENSG00000126890  ENSG00000127423  ENSG00000127564  ENSG00000127928  ENSG00000128342  ENSG00000128578  ENSG00000128610  ENSG00000128683  ENSG00000128714  ENSG00000129195  ENSG00000129654  ENSG00000129991  ENSG00000130208  ENSG00000130487  ENSG00000130513  ENSG00000130720  ENSG00000130829  ENSG00000131203  ENSG00000131747  ENSG00000131969  ENSG00000132749  ENSG00000133048  ENSG00000133063  ENSG00000133115  ENSG00000133124  ENSG00000133216  ENSG00000133466  ENSG00000133962  ENSG00000133980  ENSG00000134339  ENSG00000134538  ENSG00000135094  ENSG00000135374  ENSG00000135451  ENSG00000135480  ENSG00000135625  ENSG00000135638  ENSG00000136231  ENSG00000136881  ENSG00000136944  ENSG00000136982  ENSG00000137573  ENSG00000137745  ENSG00000137807  ENSG00000137868  ENSG00000137869  ENSG00000138083  ENSG00000138152  ENSG00000138180  ENSG00000138316  ENSG00000138346  ENSG00000138435  ENSG00000138755  ENSG00000138778  ENSG00000138798  ENSG00000139292  ENSG00000139572  ENSG00000139800  ENSG00000140511  ENSG00000140873  ENSG00000142185  ENSG00000142408  ENSG00000142945  ENSG00000143228  ENSG00000143476  ENSG00000144130  ENSG00000144395  ENSG00000145244  ENSG00000146070  ENSG00000146410  ENSG00000146670  ENSG00000146678  ENSG00000147206  ENSG00000147246  ENSG00000147381  ENSG00000147536  ENSG00000148773  ENSG00000148848  ENSG00000148965  ENSG00000149243  ENSG00000149380  ENSG00000149516  ENSG00000149948  ENSG00000149968  ENSG00000150337  ENSG00000151025  ENSG00000151224  ENSG00000151388  ENSG00000151490  ENSG00000154252  ENSG00000154451  ENSG00000154839  ENSG00000154920  ENSG00000156970  ENSG00000157193  ENSG00000157470  ENSG00000157766  ENSG00000159374  ENSG00000160161  ENSG00000160349  ENSG00000160471  ENSG00000160957  ENSG00000160973  ENSG00000162004  ENSG00000162009  ENSG00000162062  ENSG00000162344  ENSG00000162723  ENSG00000162782  ENSG00000162849  ENSG00000163283  ENSG00000163286  ENSG00000163347  ENSG00000163673  ENSG00000163739  ENSG00000163808  ENSG00000163975  ENSG00000164283  ENSG00000164362  ENSG00000164400  ENSG00000164651  ENSG00000164692  ENSG00000164694  ENSG00000164778  ENSG00000164932  ENSG00000164935  ENSG00000165164  ENSG00000165171  ENSG00000165304  ENSG00000165480  ENSG00000165490  ENSG00000165606  ENSG00000165643  ENSG00000165685  ENSG00000165891  ENSG00000166415  ENSG00000166670  ENSG00000167550  ENSG00000167580  ENSG00000167618  ENSG00000167646  ENSG00000167749  ENSG00000168065  ENSG00000168078  ENSG00000168269  ENSG00000168334  ENSG00000168542  ENSG00000168875  ENSG00000169067  ENSG00000169174  ENSG00000169245  ENSG00000169248  ENSG00000169385  ENSG00000169429  ENSG00000169495  ENSG00000169679  ENSG00000170122  ENSG00000170231  ENSG00000170369  ENSG00000170373  ENSG00000170689  ENSG00000171102  ENSG00000171208  ENSG00000171388  ENSG00000171617  ENSG00000172061  ENSG00000172232  ENSG00000172551  ENSG00000173391  ENSG00000173621  ENSG00000173894  ENSG00000174015  ENSG00000174371  ENSG00000174562  ENSG00000175832  ENSG00000175874  ENSG00000175894  ENSG00000176244  ENSG00000177238  ENSG00000177602  ENSG00000178752  ENSG00000178773  ENSG00000178776  ENSG00000179059  ENSG00000179603  ENSG00000179772  ENSG00000179934  ENSG00000180806  ENSG00000180818  ENSG00000181085  ENSG00000181143  ENSG00000181418  ENSG00000181433  ENSG00000181544  ENSG00000181577  ENSG00000182111  ENSG00000182379  ENSG00000182459  ENSG00000182492  ENSG00000182583  ENSG00000182600  ENSG00000182747  ENSG00000182870  ENSG00000183019  ENSG00000183091  ENSG00000183682  ENSG00000183734  ENSG00000183856  ENSG00000183914  ENSG00000184029  ENSG00000184661  ENSG00000184937  ENSG00000185105  ENSG00000185247  ENSG00000185686  ENSG00000186007  ENSG00000186047  ENSG00000186185  ENSG00000186193  ENSG00000186340  ENSG00000186564  ENSG00000186790  ENSG00000186871  ENSG00000187258  ENSG00000187268  ENSG00000187730  ENSG00000187741  ENSG00000188257  ENSG00000188282  ENSG00000188306  ENSG00000188610  ENSG00000189052  ENSG00000189064  ENSG00000189410  ENSG00000189433  ENSG00000196155  ENSG00000196335  ENSG00000196415  ENSG00000196460  ENSG00000196550  ENSG00000196584  ENSG00000197172  ENSG00000197472  ENSG00000197561  ENSG00000197587  ENSG00000198088  ENSG00000198443  ENSG00000198535  ENSG00000198681  ENSG00000198842  ENSG00000198930  ENSG00000203747  ENSG00000204019  ENSG00000204140  ENSG00000204542  ENSG00000204644  ENSG00000204710  ENSG00000204866  ENSG00000204936  ENSG00000205212  ENSG00000205277  ENSG00000206195  ENSG00000213401  ENSG00000213886  ENSG00000213892  ENSG00000215784  ENSG00000217236  ENSG00000221867  ENSG00000225614  ENSG00000226321  ENSG00000228630  ENSG00000229637  ENSG00000239605  ENSG00000240204  ENSG00000243709  ENSG00000244306  ENSG00000248329  ENSG00000248405  ENSG00000251151  ENSG00000253293  ENSG00000258227  ENSG00000260220  ENSG00000261587  ENSG00000261949  ENSG00000262406  ENSG00000262874  ENSG00000263513  ENSG00000264424  ENSG00000267978  ENSG00000268916  ENSG00000273706  ENSG00000274641  ENSG00000275385  ENSG00000276043  ENSG00000276085  ENSG00000277632  ENSG00000278023 | ENSG00000004776  ENSG00000004799  ENSG00000005249  ENSG00000006128  ENSG00000006747  ENSG00000007216  ENSG00000008196  ENSG00000012223  ENSG00000012504  ENSG00000013293  ENSG00000016490  ENSG00000016602  ENSG00000018236  ENSG00000018625  ENSG00000019102  ENSG00000021488  ENSG00000021645  ENSG00000022267  ENSG00000034971  ENSG00000036473  ENSG00000036672  ENSG00000036828  ENSG00000040731  ENSG00000044012  ENSG00000046653  ENSG00000048462  ENSG00000048540  ENSG00000050030  ENSG00000053328  ENSG00000053438  ENSG00000055118  ENSG00000057149  ENSG00000058866  ENSG00000059915  ENSG00000060566  ENSG00000064205  ENSG00000064309  ENSG00000065320  ENSG00000065325  ENSG00000065534  ENSG00000065609  ENSG00000066382  ENSG00000066629  ENSG00000067840  ENSG00000068615  ENSG00000068976  ENSG00000069535  ENSG00000070193  ENSG00000070808  ENSG00000071205  ENSG00000071991  ENSG00000072041  ENSG00000072133  ENSG00000072163  ENSG00000072195  ENSG00000073282  ENSG00000074211  ENSG00000074276  ENSG00000075035  ENSG00000075073  ENSG00000075673  ENSG00000076555  ENSG00000077157  ENSG00000077522  ENSG00000077943  ENSG00000078295  ENSG00000078549  ENSG00000078898  ENSG00000080224  ENSG00000080644  ENSG00000081277  ENSG00000082175  ENSG00000082293  ENSG00000084674  ENSG00000086570  ENSG00000087128  ENSG00000087258  ENSG00000088386  ENSG00000088538  ENSG00000088726  ENSG00000088926  ENSG00000089250  ENSG00000090402  ENSG00000090512  ENSG00000091128  ENSG00000091138  ENSG00000091482  ENSG00000091622  ENSG00000092009  ENSG00000092096  ENSG00000092295  ENSG00000095303  ENSG00000095637  ENSG00000095713  ENSG00000096006  ENSG00000096088  ENSG00000096395  ENSG00000099860  ENSG00000099958  ENSG00000100170  ENSG00000100191  ENSG00000100197  ENSG00000100302  ENSG00000100307  ENSG00000100314  ENSG00000100628  ENSG00000100842  ENSG00000101335  ENSG00000101605  ENSG00000101938  ENSG00000102349  ENSG00000102409  ENSG00000102539  ENSG00000102547  ENSG00000102683  ENSG00000103034  ENSG00000103241  ENSG00000104055  ENSG00000104332  ENSG00000104435  ENSG00000104722  ENSG00000104833  ENSG00000104879  ENSG00000104888  ENSG00000104936  ENSG00000105131  ENSG00000105270  ENSG00000105369  ENSG00000105398  ENSG00000105427  ENSG00000105641  ENSG00000105668  ENSG00000105675  ENSG00000105696  ENSG00000105737  ENSG00000105894  ENSG00000106018  ENSG00000106034  ENSG00000106714  ENSG00000106772  ENSG00000106809  ENSG00000107295  ENSG00000108018  ENSG00000108231  ENSG00000108242  ENSG00000108381  ENSG00000108576  ENSG00000108602  ENSG00000108823  ENSG00000108830  ENSG00000108839  ENSG00000108924  ENSG00000109101  ENSG00000109182  ENSG00000109339  ENSG00000109846  ENSG00000109906  ENSG00000109956  ENSG00000110148  ENSG00000110244  ENSG00000110245  ENSG00000111215  ENSG00000111262  ENSG00000111404  ENSG00000111405  ENSG00000111701  ENSG00000111713  ENSG00000111863  ENSG00000112183  ENSG00000112186  ENSG00000112276  ENSG00000112562  ENSG00000112818  ENSG00000112936  ENSG00000112964  ENSG00000113396  ENSG00000113430  ENSG00000113594  ENSG00000113805  ENSG00000114113  ENSG00000114200  ENSG00000114638  ENSG00000114771  ENSG00000114790  ENSG00000115361  ENSG00000115474  ENSG00000115556  ENSG00000115593  ENSG00000115665  ENSG00000115850  ENSG00000116194  ENSG00000116254  ENSG00000116741  ENSG00000116748  ENSG00000116983  ENSG00000117834  ENSG00000118094  ENSG00000118137  ENSG00000118160  ENSG00000118402  ENSG00000118407  ENSG00000118432  ENSG00000118729  ENSG00000118777  ENSG00000118898  ENSG00000119138  ENSG00000119147  ENSG00000119508  ENSG00000119715  ENSG00000119938  ENSG00000120057  ENSG00000120251  ENSG00000120471  ENSG00000120729  ENSG00000121207  ENSG00000121413  ENSG00000121440  ENSG00000121552  ENSG00000121577  ENSG00000121742  ENSG00000121871  ENSG00000121898  ENSG00000122012  ENSG00000122121  ENSG00000122367  ENSG00000122585  ENSG00000122756  ENSG00000123119  ENSG00000123243  ENSG00000123560  ENSG00000123570  ENSG00000124143  ENSG00000124440  ENSG00000124466  ENSG00000124701  ENSG00000124749  ENSG00000124939  ENSG00000125285  ENSG00000125740  ENSG00000125780  ENSG00000125851  ENSG00000125998  ENSG00000126010  ENSG00000126233  ENSG00000126500  ENSG00000126549  ENSG00000126803  ENSG00000126878  ENSG00000126950  ENSG00000127129  ENSG00000127241  ENSG00000127472  ENSG00000127951  ENSG00000128040  ENSG00000128510  ENSG00000128573  ENSG00000128591  ENSG00000128594  ENSG00000128849  ENSG00000129151  ENSG00000129194  ENSG00000129214  ENSG00000129244  ENSG00000129596  ENSG00000130037  ENSG00000130055  ENSG00000130176  ENSG00000130226  ENSG00000130234  ENSG00000130643  ENSG00000130822  ENSG00000130957  ENSG00000131094  ENSG00000131471  ENSG00000131482  ENSG00000131668  ENSG00000131730  ENSG00000132164  ENSG00000132464  ENSG00000132465  ENSG00000132517  ENSG00000132563  ENSG00000132639  ENSG00000132744  ENSG00000132840  ENSG00000132855  ENSG00000132938  ENSG00000133107  ENSG00000133328  ENSG00000133392  ENSG00000133687  ENSG00000133710  ENSG00000133742  ENSG00000133800  ENSG00000133878  ENSG00000133985  ENSG00000134020  ENSG00000134121  ENSG00000134201  ENSG00000134216  ENSG00000134443  ENSG00000134531  ENSG00000134533  ENSG00000134548  ENSG00000134551  ENSG00000134757  ENSG00000134760  ENSG00000134762  ENSG00000134812  ENSG00000134873  ENSG00000134917  ENSG00000135046  ENSG00000135218  ENSG00000135298  ENSG00000135333  ENSG00000135406  ENSG00000135424  ENSG00000135447  ENSG00000135773  ENSG00000135842  ENSG00000136002  ENSG00000136155  ENSG00000136267  ENSG00000136274  ENSG00000136457  ENSG00000136546  ENSG00000136689  ENSG00000136694  ENSG00000136695  ENSG00000136696  ENSG00000136842  ENSG00000136872  ENSG00000137094  ENSG00000137265  ENSG00000137273  ENSG00000137726  ENSG00000137975  ENSG00000138075  ENSG00000138109  ENSG00000138271  ENSG00000138308  ENSG00000138311  ENSG00000138356  ENSG00000138650  ENSG00000138685  ENSG00000138722  ENSG00000138823  ENSG00000138944  ENSG00000139200  ENSG00000139910  ENSG00000139973  ENSG00000139988  ENSG00000140254  ENSG00000140287  ENSG00000140459  ENSG00000140465  ENSG00000140519  ENSG00000140600  ENSG00000140682  ENSG00000141052  ENSG00000141161  ENSG00000141338  ENSG00000141434  ENSG00000141579  ENSG00000141622  ENSG00000141639  ENSG00000142583  ENSG00000142623  ENSG00000142959  ENSG00000142973  ENSG00000143171  ENSG00000143196  ENSG00000143318  ENSG00000143320  ENSG00000143502  ENSG00000143536  ENSG00000143546  ENSG00000143595  ENSG00000143631  ENSG00000143816  ENSG00000143869  ENSG00000143921  ENSG00000144031  ENSG00000144035  ENSG00000144191  ENSG00000144218  ENSG00000144230  ENSG00000144331  ENSG00000144339  ENSG00000144712  ENSG00000144834  ENSG00000144847  ENSG00000144891  ENSG00000145248  ENSG00000145283  ENSG00000145362  ENSG00000145384  ENSG00000145626  ENSG00000145687  ENSG00000145700  ENSG00000145721  ENSG00000145777  ENSG00000145861  ENSG00000145879  ENSG00000145936  ENSG00000146013  ENSG00000146122  ENSG00000146151  ENSG00000146267  ENSG00000146352  ENSG00000146469  ENSG00000146755  ENSG00000147166  ENSG00000147576  ENSG00000147588  ENSG00000147606  ENSG00000147655  ENSG00000147697  ENSG00000147724  ENSG00000148483  ENSG00000148798  ENSG00000148826  ENSG00000149021  ENSG00000149294  ENSG00000149451  ENSG00000149575  ENSG00000149591  ENSG00000149596  ENSG00000149970  ENSG00000150625  ENSG00000150627  ENSG00000150672  ENSG00000150764  ENSG00000151320  ENSG00000151892  ENSG00000152137  ENSG00000152208  ENSG00000152217  ENSG00000152578  ENSG00000152580  ENSG00000152785  ENSG00000153002  ENSG00000153086  ENSG00000153446  ENSG00000153802  ENSG00000153822  ENSG00000153902  ENSG00000154080  ENSG00000154165  ENSG00000154175  ENSG00000154227  ENSG00000154258  ENSG00000154269  ENSG00000154330  ENSG00000154553  ENSG00000154556  ENSG00000154645  ENSG00000154646  ENSG00000154678  ENSG00000154721  ENSG00000154734  ENSG00000155761  ENSG00000155816  ENSG00000155918  ENSG00000155970  ENSG00000156113  ENSG00000156218  ENSG00000156222  ENSG00000156284  ENSG00000156395  ENSG00000156687  ENSG00000156689  ENSG00000156920  ENSG00000157005  ENSG00000157017  ENSG00000157404  ENSG00000157445  ENSG00000157551  ENSG00000157927  ENSG00000158055  ENSG00000158246  ENSG00000158445  ENSG00000158458  ENSG00000158516  ENSG00000158560  ENSG00000158764  ENSG00000158865  ENSG00000159197  ENSG00000159224  ENSG00000159251  ENSG00000159307  ENSG00000159337  ENSG00000159387  ENSG00000159388  ENSG00000159516  ENSG00000159527  ENSG00000160097  ENSG00000160181  ENSG00000160182  ENSG00000160307  ENSG00000160801  ENSG00000160868  ENSG00000161281  ENSG00000161640  ENSG00000161649  ENSG00000162040  ENSG00000162373  ENSG00000162374  ENSG00000162398  ENSG00000162409  ENSG00000162460  ENSG00000162461  ENSG00000162614  ENSG00000162706  ENSG00000162761  ENSG00000162951  ENSG00000162998  ENSG00000163017  ENSG00000163141  ENSG00000163145  ENSG00000163202  ENSG00000163207  ENSG00000163209  ENSG00000163216  ENSG00000163218  ENSG00000163220  ENSG00000163273  ENSG00000163295  ENSG00000163328  ENSG00000163331  ENSG00000163377  ENSG00000163380  ENSG00000163394  ENSG00000163431  ENSG00000163531  ENSG00000163581  ENSG00000163586  ENSG00000163623  ENSG00000163629  ENSG00000163637  ENSG00000163687  ENSG00000163710  ENSG00000163815  ENSG00000163873  ENSG00000163884  ENSG00000163959  ENSG00000164089  ENSG00000164107  ENSG00000164120  ENSG00000164122  ENSG00000164270  ENSG00000164303  ENSG00000164326  ENSG00000164406  ENSG00000164418  ENSG00000164520  ENSG00000164530  ENSG00000164619  ENSG00000164764  ENSG00000164816  ENSG00000164822  ENSG00000164825  ENSG00000164850  ENSG00000165072  ENSG00000165186  ENSG00000165192  ENSG00000165197  ENSG00000165300  ENSG00000165323  ENSG00000165349  ENSG00000165410  ENSG00000165449  ENSG00000165495  ENSG00000165553  ENSG00000165794  ENSG00000165799  ENSG00000165821  ENSG00000165966  ENSG00000165995  ENSG00000165996  ENSG00000166165  ENSG00000166183  ENSG00000166268  ENSG00000166292  ENSG00000166317  ENSG00000166391  ENSG00000166402  ENSG00000166405  ENSG00000166407  ENSG00000166482  ENSG00000166535  ENSG00000166819  ENSG00000166828  ENSG00000166831  ENSG00000166863  ENSG00000166959  ENSG00000167281  ENSG00000167549  ENSG00000167641  ENSG00000167653  ENSG00000167654  ENSG00000167656  ENSG00000167676  ENSG00000167759  ENSG00000167768  ENSG00000167769  ENSG00000167779  ENSG00000167800  ENSG00000167916  ENSG00000168060  ENSG00000168079  ENSG00000168081  ENSG00000168267  ENSG00000168309  ENSG00000168447  ENSG00000168477  ENSG00000168481  ENSG00000168497  ENSG00000168546  ENSG00000168702  ENSG00000168748  ENSG00000168874  ENSG00000168903  ENSG00000168913  ENSG00000169083  ENSG00000169085  ENSG00000169252  ENSG00000169340  ENSG00000169418  ENSG00000169469  ENSG00000169474  ENSG00000169509  ENSG00000169550  ENSG00000169583  ENSG00000169594  ENSG00000169605  ENSG00000169760  ENSG00000169903  ENSG00000169906  ENSG00000170011  ENSG00000170153  ENSG00000170271  ENSG00000170298  ENSG00000170323  ENSG00000170419  ENSG00000170423  ENSG00000170426  ENSG00000170465  ENSG00000170476  ENSG00000170477  ENSG00000170482  ENSG00000170500  ENSG00000170577  ENSG00000171094  ENSG00000171234  ENSG00000171243  ENSG00000171246  ENSG00000171303  ENSG00000171346  ENSG00000171401  ENSG00000171431  ENSG00000171517  ENSG00000171533  ENSG00000171711  ENSG00000171819  ENSG00000171873  ENSG00000171885  ENSG00000171916  ENSG00000171954  ENSG00000172005  ENSG00000172020  ENSG00000172247  ENSG00000172260  ENSG00000172348  ENSG00000172382  ENSG00000172403  ENSG00000172425  ENSG00000172461  ENSG00000172478  ENSG00000172548  ENSG00000172689  ENSG00000172782  ENSG00000172915  ENSG00000172935  ENSG00000172987  ENSG00000173175  ENSG00000173212  ENSG00000173237  ENSG00000173406  ENSG00000173597  ENSG00000173641  ENSG00000174099  ENSG00000174226  ENSG00000174236  ENSG00000174358  ENSG00000174460  ENSG00000174514  ENSG00000174564  ENSG00000174576  ENSG00000174611  ENSG00000174944  ENSG00000174992  ENSG00000175084  ENSG00000175121  ENSG00000175161  ENSG00000175356  ENSG00000175497  ENSG00000175785  ENSG00000175906  ENSG00000175984  ENSG00000176194  ENSG00000176399  ENSG00000176533  ENSG00000176928  ENSG00000176956  ENSG00000177301  ENSG00000177363  ENSG00000177511  ENSG00000177519  ENSG00000178031  ENSG00000178233  ENSG00000178363  ENSG00000178597  ENSG00000178690  ENSG00000178919  ENSG00000178934  ENSG00000179094  ENSG00000179520  ENSG00000179593  ENSG00000179639  ENSG00000179915  ENSG00000179954  ENSG00000180155  ENSG00000180251  ENSG00000180660  ENSG00000180871  ENSG00000181072  ENSG00000181092  ENSG00000181195  ENSG00000181541  ENSG00000181778  ENSG00000181856  ENSG00000182040  ENSG00000182103  ENSG00000182156  ENSG00000182175  ENSG00000182253  ENSG00000182333  ENSG00000182575  ENSG00000182585  ENSG00000182836  ENSG00000182916  ENSG00000182938  ENSG00000182983  ENSG00000183034  ENSG00000183036  ENSG00000183134  ENSG00000183230  ENSG00000183347  ENSG00000183454  ENSG00000183578  ENSG00000183607  ENSG00000183715  ENSG00000183783  ENSG00000183798  ENSG00000183963  ENSG00000184144  ENSG00000184226  ENSG00000184502  ENSG00000184702  ENSG00000184811  ENSG00000184905  ENSG00000185046  ENSG00000185345  ENSG00000185352  ENSG00000185432  ENSG00000185437  ENSG00000185479  ENSG00000185681  ENSG00000185873  ENSG00000185966  ENSG00000186009  ENSG00000186081  ENSG00000186115  ENSG00000186198  ENSG00000186297  ENSG00000186462  ENSG00000186474  ENSG00000186510  ENSG00000186642  ENSG00000186766  ENSG00000186806  ENSG00000186832  ENSG00000186847  ENSG00000186868  ENSG00000187054  ENSG00000187134  ENSG00000187140  ENSG00000187288  ENSG00000187479  ENSG00000187689  ENSG00000187714  ENSG00000187848  ENSG00000187957  ENSG00000188001  ENSG00000188100  ENSG00000188153  ENSG00000188293  ENSG00000188373  ENSG00000188505  ENSG00000188508  ENSG00000188611  ENSG00000188730  ENSG00000188738  ENSG00000188778  ENSG00000188828  ENSG00000189001  ENSG00000189051  ENSG00000189129  ENSG00000189134  ENSG00000189367  ENSG00000189377  ENSG00000196104  ENSG00000196263  ENSG00000196344  ENSG00000196376  ENSG00000196482  ENSG00000196542  ENSG00000196549  ENSG00000196557  ENSG00000196616  ENSG00000196620  ENSG00000196660  ENSG00000196754  ENSG00000196805  ENSG00000197165  ENSG00000197191  ENSG00000197353  ENSG00000197361  ENSG00000197380  ENSG00000197565  ENSG00000197576  ENSG00000197584  ENSG00000197632  ENSG00000197641  ENSG00000197766  ENSG00000197888  ENSG00000198074  ENSG00000198092  ENSG00000198099  ENSG00000198467  ENSG00000198483  ENSG00000198523  ENSG00000198576  ENSG00000198597  ENSG00000198624  ENSG00000198807  ENSG00000198838  ENSG00000198932  ENSG00000198947  ENSG00000203685  ENSG00000203722  ENSG00000203785  ENSG00000203786  ENSG00000203867  ENSG00000204323  ENSG00000204421  ENSG00000204544  ENSG00000204653  ENSG00000204740  ENSG00000204872  ENSG00000204950  ENSG00000204978  ENSG00000205002  ENSG00000205038  ENSG00000205221  ENSG00000205363  ENSG00000205364  ENSG00000205420  ENSG00000205795  ENSG00000206072  ENSG00000206073  ENSG00000206579  ENSG00000211445  ENSG00000212900  ENSG00000213088  ENSG00000213366  ENSG00000213759  ENSG00000213760  ENSG00000213996  ENSG00000214575  ENSG00000214711  ENSG00000214782  ENSG00000215218  ENSG00000215853  ENSG00000218416  ENSG00000226306  ENSG00000227051  ENSG00000227471  ENSG00000228314  ENSG00000229183  ENSG00000229859  ENSG00000233670  ENSG00000237125  ENSG00000237515  ENSG00000239474  ENSG00000240505  ENSG00000240771  ENSG00000241224  ENSG00000241635  ENSG00000241794  ENSG00000243244  ENSG00000243284  ENSG00000243955  ENSG00000244067  ENSG00000244094  ENSG00000244122  ENSG00000244734  ENSG00000245105  ENSG00000248485  ENSG00000249948  ENSG00000253250  ENSG00000254709  ENSG00000256162  ENSG00000256713  ENSG00000256812  ENSG00000257335  ENSG00000259417  ENSG00000260230  ENSG00000261272  ENSG00000265190  ENSG00000266200  ENSG00000266524  ENSG00000266964  ENSG00000269855  ENSG00000269964  ENSG00000273777  ENSG00000277586  ENSG00000278505 |
