# Supplementary material for: Could microtubule inhibitors be the best choice of therapy in gastric cancer with high immune activity: mutant DYNC1H1 as a biomarker
Source: Aging (Albany NY). 2020 Nov 20;12(24):25101–19. doi: 10.18632/aging.104084 (PMC7803585; doi:10.18632/aging.104084)
Supplement: Supplementary Table 2 [file aging-12-104084-s003.pdf]

SUPPLEMENTARY TABLE

Supplementary Table 2. The functional gene sets of each drug type from molecular signatures database.

Candidate drug types	Functional gene sets
1. Microtubule inhibitor	BIOCARTA_G2_PATHWAY
	BIOCARTA_MTOR_PATHWAY
	BIOCARTA_NFKB_PATHWAY
	GO_MICROTUBULE
	GO_MICROTUBULE_BINDING
	GO_MICROTUBULE_DEPOLYMERIZATION
	GO_MICROTUBULE_NUCLEATION
	GO_MICROTUBULE_POLYMERIZATION
	GO_MICROTUBULE_POLYMERIZATION_OR_DEPOLYMERIZATION
	GO_REGULATION_OF_MICROTUBULE_POLYMERIZATION_OR_DEPOLYMERIZATION
	GO_S_ADENOSYLHOMOCYSTEINE_METABOLIC_PROCESS
	MICROTUBULE
	MICROTUBULE_BINDING
	MICROTUBULE_POLYMERIZATION_OR_DEPOLYMERIZATION
	REACTOME_POST_CHAPERONIN_TUBULIN_FOLDING_PATHWAY
	REACTOME_FORMATION_OF_TUBULIN_FOLDING_INTERMEDIATES_BY_CCT_TRIC
2. Corticosteroid agonist	GO_CELLULAR_RESPONSE_TO_CORTICOSTEROID_STIMULUS
	GO_REGULATION_OF_CORTICOSTEROID_HORMONE_SECRETION
	GO_RESPONSE_TO_CORTICOSTEROID
	GO_RESPONSE_TO_CORTICOSTERONE
3. Androgen receptor agonist	GO_ANDROGEN_RECEPTOR_BINDING
	GO_ANDROGEN_RECEPTOR_SIGNALING_PATHWAY
	GO_NEGATIVE_REGULATION_OF_ANDROGEN_RECEPTOR_SIGNALING_PATHWAY
	GO_REGULATION_OF_ANDROGEN_RECEPTOR_SIGNALING_PATHWAY
	GO_TESTOSTERONE_BIOSYNTHETIC_PROCESS
4. Protein synthesis inhibitor	GO_POLYSOME
	GO_RIBOSOMAL_SMALL_SUBUNIT_BIOGENESIS
	GO_RIBOSOMAL_LARGE_SUBUNIT_BIOGENESIS
	GO_RIBOSOME_BIOGENESIS
	GO_SIGNAL_RECOGNITION_PARTICLE_ENDOPLASMIC_RETICULUM_TARGETING
	GO_TRNA_CATABOLIC_PROCESS
	GO_TRNA_METABOLIC_PROCESS
	GO_EUKARYOTIC_TRANSLATION_INITIATION_FACTOR_3_COMPLEX
5. Cyclooxygenase inhibitor	0
6. Leukotriene receptor antagonist	GO_LEUKOTRIENE_D4_BIOSYNTHETIC_PROCESS
7. Adrenergic receptor antagonist	GO_ALPHA_2A_ADRENERGIC_RECEPTOR_BINDING
	GO_ADRENERGIC_RECEPTOR_BINDING
	GO_ADRENERGIC_RECEPTOR_SIGNALING_PATHWAY
	GO_BETA_2_ADRENERGIC_RECEPTOR_BINDING

GO_NEGATIVE_REGULATION_OF_ADENYLATE_CYCLASE_ACTIVATING_ADRENERGIC_RECEPTOR_SIGNALING_PATHWAY
